# Evolution of Hip Muscles Strength in Femoroacetabular Impingement Patients Treated by Arthroscopy or Surgical Hip Dislocation: A Retrospective Exploratory Study

**DOI:** 10.3390/biology11121765

**Published:** 2022-12-06

**Authors:** Guillaume Servant, François Fourchet, Anthony Pernoud, Hugo Bothorel, Panayiotis Christofilopoulos

**Affiliations:** 1 Physiotherapy Department and Motion Analysis Lab, Swiss Olympic Medical Center, La Tour Hospital, CH-1217 Meyrin, Switzerland; 2 Research Department, La Tour Hospital, CH-1217 Meyrin, Switzerland; 3 Orthopedic Department, La Tour Hospital, CH-1217 Meyrin, Switzerland

**Keywords:** femoroacetabular impingement syndrome, FAI, arthroscopy, surgical hip dislocation, SHD, hip muscles strength, rehabilitation, physiotherapy

## Abstract

**Simple Summary:**

Femoroacetabular impingement represents an important burden for affected patients in their daily life. Possible and successful treatments to alleviate patient symptoms are corrections of bone deformities using either arthroscopy or surgical hip dislocation. Nevertheless, the aforementioned surgeries might also weaken the operated hip in addition to the impact of the pathology itself. There is, however, little or no published data on the impact of arthroscopy and surgical hip dislocation on hip muscles strength, which motivated us to perform this study. For arthroscopy, we found that patients exhibited on the operated hip a moderate decrease in abductors strength, as well as a small but noticeable decrease in hamstrings, external rotators and flexors strengths three months after surgery. Interestingly, patients also had a small but relevant strength decrease on the non-operated side, located on external rotators. For surgical hip dislocation, patients exhibited on the operated hip a large decrease in internal rotators strength and a moderate decrease in abductors, quadriceps and external rotators strengths. These findings suggest that several hip muscles can be moderately or largely affected after arthroscopy (abductors) or surgical hip dislocation (internal and external rotators, abductors and quadriceps). This study also suggests that a rehabilitation method based on isolated muscle reinforcement and functional exercises is needed and emphasizes the need for a rehabilitation protocol that goes beyond three postoperative months.

**Abstract:**

Hip arthroscopy and surgical hip dislocation (SHD) can be adequate surgical options for patients suffering from femoroacetabular impingement (FAI) syndrome, but there is to date no published data on their impact on hip muscles strength. The purpose of this retrospective study was, therefore, to evaluate it on a consecutive series of 50 FAI patients treated either by arthroscopy (n = 29, aged 27.4 ± 7.5 years, 76% of women) or SHD (n = 21, aged 25.9 ± 6.5 years, 38% of women) at La Tour Hospital between 2020 and 2021. The bilateral isometric strengths of eight hip-related muscles were evaluated before and three months after surgery (halfway through the rehabilitation program). For arthroscopy, a statistically significant (*p* < 0.05) reduction in hip muscles strength could be noted on the operated hamstrings (1.49 ± 0.43 vs. 1.39 ± 0.38 Nm/kg), flexors (1.88 ± 0.46 vs. 1.73 ± 0.41 Nm/kg), abductors (1.97 ± 0.42 vs. 1.72 ± 0.40 Nm/kg) and external rotators (1.17 ± 0.40 vs. 1.04 ± 0.37 Nm/kg). The abductors were the most affected muscles, with 45% of the patients suffering from a strength reduction ≥15%. The non-operated external rotators were also affected but to a lesser extent (1.21 ± 0.38 vs. 1.10 ± 0.36 Nm/kg). For SHD, a statistically significant strength reduction could be noted on the operated extensors (2.28 ± 0.84 vs. 2.05 ± 0.70 Nm/kg), abductors (1.87 ± 0.49 vs. 1.65 ± 0.41 Nm/kg), quadriceps (2.96 ± 0.92 vs. 2.44 ± 0.89 Nm/kg), external rotators (1.16 ± 0.42 vs. 0.93 ± 0.36 Nm/kg) and internal rotators (1.26 ± 0.38 vs. 0.96 ± 0.30 Nm/kg). The internal rotators were the most affected muscles, with 75% of the patients suffering from a strength reduction ≥15%. To conclude, particular attention should be paid to operated abductors for patients treated by arthroscopy as well as operated internal/external rotators, abductors and quadriceps for those treated by surgical hip dislocation. It reinforces that a rehabilitation method based on isolated muscle reinforcement and functional exercises that goes beyond three postoperative months is needed.

## 1. Introduction

Femoroacetabular impingement syndrome (FAI) is often reported as the most common cause of hip pain, with an estimated prevalence of 10% in the general population [1]. Such symptoms can be reported by patients presenting bony structural disorders of the hip, which trigger premature contact between the proximal femur and the acetabulum [2,3]. Different FAI types have already been described involving either a cam morphology, which results from a loss of sphericity at the femoral head–neck junction, a pincer morphology characterized by an abnormal prominence of the acetabular rim on the anterolateral side, or both [2,3]. The repetition of bony impacts and associated pain considerably reduce patient hip range of motion and muscle strength, thereby decreasing functional daily activity and increasing risks of hip osteoarthritis in the long-term due to tissue and cartilage injuries [4,5,6].

Conservative and pharmacological treatments can be tempted initially but remain sometimes insufficient to fully alleviate patient symptoms [7]. Under those circumstances, a surgical option might be needed to directly treat the underlying bone deformities, either by arthroscopy or surgical hip dislocation (SHD). Nowadays, arthroscopy is often preferred over SHD since it offers a minimally invasive procedure, faster rehabilitation, minor soft tissue damage and very satisfactory outcomes [7,8,9]. However, the shift away from SHD in decision-making for treatment of FAI should be made with caution since the SHD technique can provide superior results for patients with important bone deformities through greater joint access and dynamic assessment of bony corrections [10,11].

While many authors reported improvement in patients’ quality of life and functional status following arthroscopy using patient-reported outcome measures (PROMs) [2,12,13,14,15], only a few studies investigated the surgical impact on hip muscles strength [12,16,17]. Furthermore, correction of bone deformities following SHD goes along with soft tissue damage and trochanteric osteotomy, which implies longer patient recovery [11,18]. The rehabilitation programs are, therefore, of great importance and can be guided by assessments of patient functional status, such as range of motion, specific tests and bilateral strength evaluation. Hip muscle strength in FAI patients is a topic of interest since it is reduced by the pathology itself [19,20,21] and additionally affected by surgery [12,16,17]. Noteworthily, muscle strength is one of the last clinical parameters measured in common practice, although it has been reported as the most adequate proxy for patient functional progression and surgical success [22]. Consequently, the purpose of the present study was to evaluate the bilateral changes in hip muscles strength following arthroscopy and SHD for treatment of FAI syndrome just before the functional restoration phase of the rehabilitation program.

## 2. Materials and Methods

The authors retrospectively evaluated a consecutive series of 50 patients treated by either arthroscopy (n = 29) or SHD (n = 21) for FAI at La Tour Hospital from March 2020 to September 2021. All patients were rigorously evaluated before and after surgery at the Motion Analysis Laboratory of the physiotherapy department. Patients treated by arthroscopy were aged 27.4 ± 7.5 at index surgery and comprised 22 women (76%) and 7 men (24%). Patients treated by SHD were aged 25.9 ± 6.5 years (range, 16–36) at index surgery and comprised 13 men (62%) and 8 women (38%). None of the patients were excluded because of the following a priori defined exclusion criteria: pregnancy, previous lower extremity surgeries, arthritis, stroke, spinal cord lesion or injury, head trauma, peripheral diabetes or any other type of neurological disease that could influence the nervous system or signs of severe osteoarthritis. All patients presented a cam or pincer morphology, or a mix of both with or without labral lesions. One patient refused to participate in this study through use of his data and was, therefore, excluded from the SHD group. All patients were operated on by the same senior surgeon (PC) at La Tour Hospital following the same arthroscopic technique detailed below. Since this study is exploratory and based on clinical data that are routinely collected at our institution to evaluate patients’ clinical improvement, a priori approval from our ethical committee was not required. However, all the patients included in this study gave their written informed consent for the use of their data in research projects.

### 2.1. Pre- and Postoperative Hip Muscles Strength Assessment

**Figure 1:**
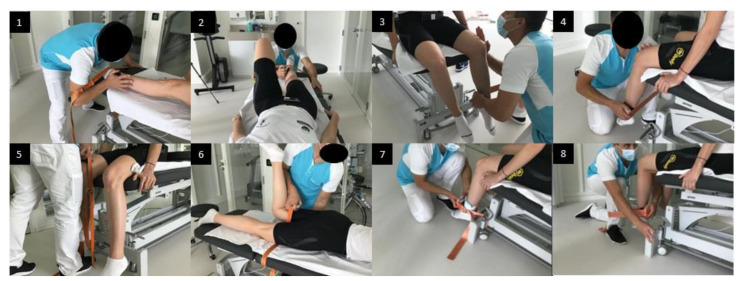
Hip-related muscle groups strength testing positions. (1) Hip abductors, (2) Hip adductors, (3) Hip external rotators, (4) Hip internal rotators, (5) Hip flexors, (6) Hip extensors, (7) Quadriceps, (8) Hamstrings. Further explanations can be found in Supplementary Materials.

The isometric muscles strengths of both hips (maximal voluntary contraction, MVC) were evaluated before surgery and at 3 postoperative months using a handheld dynamometer (Hoggan MicroFET2, Scientific L.L.C., Salt Lake City, UT, USA) with a sampling frequency of 100 Hz [23]. The measures concerned eight hip-related muscle groups: (1) abductors, (2) adductors, (3) flexors, (4) extensors, (5) external rotators, (6) internal rotators, (7) quadriceps and (8) hamstrings. All evaluations were performed following a strict methodology by a senior physiotherapist (GS). Patients were evaluated after a 6 min warm-up on a stationary bike in different testing positions, as described by Thorborg et al. in 2013 (Figure 1) [24]. According to Thorborg et al.’s recommendations, the subjects stabilized themselves by holding the examination table whilst a fixation-belt was used in order to obtain better test–retest reliability [24]. After explaining procedures, three isometric maximum voluntary contractions of 6 s, separated by 30 s of rest, were performed on each muscle group under verbal encouragement. The highest value of the three repetitions was recorded. If the last measurement was the highest, another measurement was conducted until no further force increase was measured. Peak forces were measured in Newton and then normalized by the arm-lever (in meters) and by the body weight (kilogram) in order to be in Nm/kg unit.

### 2.2. Surgical Technique—Arthroscopy

Patients were placed in a supine position on a traction table. Both lower limbs were placed in traction for a variable amount of time and standard disinfection/draping was performed. The procedure was performed using portals on the AL (anterolateral), AAL (anterior–anterolateral), MAP (medial–anterior) sides and an inter-portal on the AL–MAP sides. For the central compartment, the statuses of the cartilage and round ligament were checked for any sign of injury or disinsertion that could lead to debridement and reinsertion using anchors. The presence of possible synovitis was also checked, and, if positive, led to synovectomy with capsular preservation. A plasty of the anteroinferior iliac spine (AIIS) was performed (n = depending on its morphology type). An acetabuloplasty was also performed depending on the FAI type. Once these procedures were completed, the traction was released and an L-shaped capsulotomy keeping intact the medial limb of the iliofemoral ligament was performed. Then, the hip was tested in flexion, which must be free of impingement between the acetabular rim and the femoral neck. Internal flexion-rotation should also be conflict-free. Regarding the peripheral compartment, an inspection of the femoral head was performed to assess the presence of FAI clinical signs, such as a large bump at the head–neck junction or filling of the lateral and antero-lateral parts of the cervico-cephalic junction. For these cases, osteochondroplasty of the head–neck junction was performed while respecting the retinacular vessels. The vertical capsulotomy was repaired with absorbable sutures, and a clinical and radiological check-up was finally performed followed by a classical closure with Prolene sutures.

### 2.3. Surgical Technique—Surgical Hip Dislocation (SHD)

Patients were placed under general anesthesia and positioned in lateral decubitus. Antibiotic prophylaxis was administered, followed by disinfection and draping of the entire operated lower limb. A Gauthier approach was used with a Z-shaped trochanteric osteotomy, followed by detachment of the gluteus minimus from the joint capsule using a Z-shaped capsulotomy. The hip was dislocated in external flexion-rotation and an inspection of the central compartment was performed to look for possible labral or articular cartilage instability. The status of the acetabular and cephalic cartilage was also checked. An acetabuloplasty was then performed and the labrum was reinserted using a variable number of JuggerKnot anchors. A plasty of the anterior inferior iliac spine (AIIS) was performed in 16 patients (80%) if it was protruding and impinging on the femoral neck while limiting internal rotation. After this procedure, the degree of flexion and internal rotation were checked so that they, respectively, exceed 100° and 30°.The femoral head was then examined for clinical signs of FAI, such as bulging on the head–neck junction and filling of the anterior and antero-lateral parts of the cervico-cephalic junction. The round ligament was excised and then an osteochondroplasty of the head–neck junction was performed while respecting the retinacular vessels. The hip was thereafter reduced and its stability as well as correct mobility were verified (internal rotation in flexion should be around 30°). An abundant lavage was performed, followed by closure of the joint capsule and osteosynthesis of the greater trochanter with two 4.5 mm screws. Radiological monitoring was performed intraoperatively to confirm good reduction in acetabular coverage and adequate osteosynthesis of the greater trochanter. Finally, an abundant lavage was repeated before closing the incision using a redon drainage and surgical staples.

### 2.4. Postoperative Patient Rehabilitation

The supervised rehabilitation protocol started on the intervention day a few hours after surgery in accordance with the 2019 International Society for Hip Arthroscopy (ISHA) convention.

#### 2.4.1. First Stage—Immediate Postoperative

During hospitalization, patients walked using crutches with 15 kg partial weight bearing on the operated limb. Three or four days after surgery, the patients went home and were asked to keep using a continuous motion device.

#### 2.4.2. Second Stage—Early Impairment

The second stage started 10 days following surgery when the scar healing allowed the patients to go into water. Nine hydrotherapy group sessions were then performed into a pool to mobilize the tissues and facilitate the kinematics of the hip while paying attention to the patients’ constraints and mobilization limitations. A land-based physiotherapy session was added between the fourth and fifth hydrotherapy sessions to explain exercises to be performed independently at home. A booklet was then distributed at the end of the session to help patients reproduce correctly the aforementioned exercises.

#### 2.4.3. Third Stage—Late Impairment

Full weight-bearing is then progressively allowed in a third stage but adapted to patient pain. Specific attention was, therefore, paid to the gait pattern and hip muscles voluntary contractions. This third stage also comprised bi-weekly individual sessions of 30 min to closely follow the evolution of patients’ symptoms until the functional test at 3 postoperative months. Although the present study is based on the data obtained at this specific time-point, it is worth mentioning that the rehabilitation lasts almost 7 months with the following phase.

#### 2.4.4. Fourth Stage—Functional Restoration

A progressive load is then applied during the fourth and last stage to increase hip muscle strength, endurance, function, dynamic balance and gait pattern. Progressive and adapted physical activities are recommended and manual therapy techniques are used to improve hip range of motion and reduce pain [25,26,27,28,29]. Rehabilitation is finally completed with a phase of increased muscle strength based on heavy load exercises and return to full function of the hip. An additional stage of return to sports activity until return to pre-symptomatic performance (RTP) if necessary is also carried out, which generally lasts 1 to 3 months according to patient characteristics.

### 2.5. Sample Size Calculations and Statistical Analyses

For arthroscopy, Beck et al. recently published that preoperative hip extension strength was an important predictor of achieving a postoperative patient acceptable symptom state [30]. Furthermore, the extensors peak force for operated hips was reported to be 2.97 ± 0.83 Nm/kg in FAI patients [12], and a difference of 15% in muscle strength appears to be clinically relevant since it has been used in a sample size calculation for a comparable study [16]. Based on the aforementioned findings, 29 FAI patients would be required to significantly detect a 15% difference in extensors MVC on operated hips with a statistical power of 0.80 and a significant alpha level of 0.05.

For SHD, a residual abductor weakness has been already reported as a potential complication [31,32]. Casartelli et al. [19] reported an abductor strength of 1.81 ± 0.43 Nm/kg in non-operated FAI patients, while a change in muscle strength of 15% seems to be clinically relevant [16]. Based on the aforementioned findings, 20 FAI patients would be required to significantly detect a 15% difference in abductors MVC on operated hips with a statistical power of 0.80 and a significant alpha level of 0.05.

Descriptive statistics were used to summarize the data. Continuous variables were reported as mean ± standard deviation, interquartile range (IQR) and minimum–maximum values, while categorical data were reported as proportions. The normality of continuous variable distributions was assessed by the Shapiro–Wilk test. Muscle strength comparisons between different time-points as well as between operated and non-operated hips were also conducted using Wilcoxon signed rank tests or paired Student’s *t*-tests. The effect size of the treatment was calculated using Hedges’ g for the different studied outcomes and interpreted as follows: small (0.2 ≤ Hedges’ g < 0.5), medium (0.5 ≤ Hedges’ g < 0.8) and large (0.8 ≤ Hedges’ g < 1.2) [33]. The correlations between hip muscles strength reduction on the operated and non-operated sides were analyzed using Pearson’s coefficients, reported with 95% confidence interval (95%IC) and interpreted as negligible (r = 0.00 to 0.09), weak (r = 0.10 to 0.39), moderate (r = 0.40 to 0.69), strong (r = 0.70 to 0.89) or very strong (r = 0.90 to 1.00) [34]. The analyses were performed using R (version 3.6.2, R Foundation for Statistical Computing, Vienna, Austria), and *p*-values < 0.05 were considered significant.

## 3. Results

### 3.1. Arthroscopy

None of the included patients experienced a complication during surgery or during the three following months. The operated and non-operated hips exhibited comparable muscle strength before surgery (Table 1).

**Figure 2:**
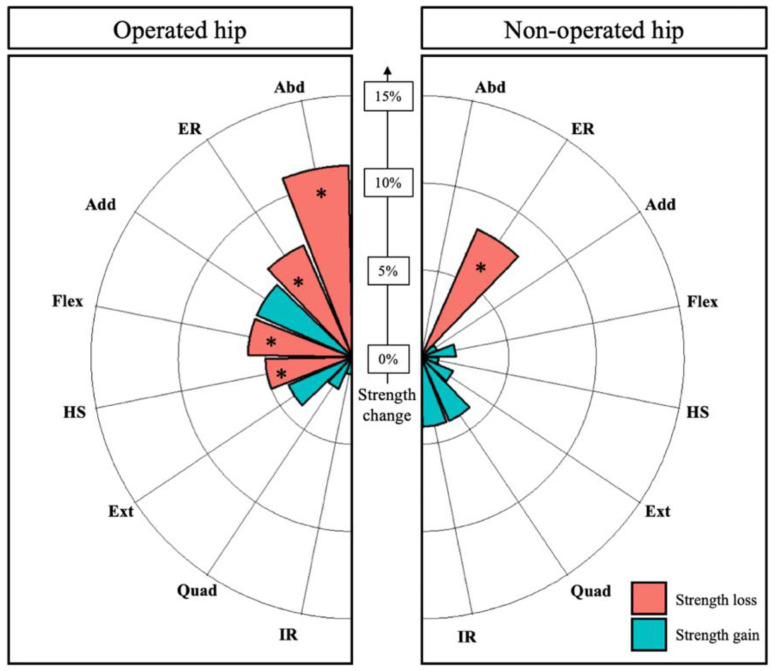
Pre- to postoperative changes in muscle strength (%) on the operated and non-operated hips. * indicates a statistically significant variation. Internal rotators (IR), external rotators (ER), quadriceps (Quad), abductors (Abd), extensor (Ext), hamstrings (HS), flexors (Fl), adductors (Add).

The effect size (Hedges’ g) of surgery on muscle strength changes was small for operated hamstrings (−0.25), external rotators (−0.32), flexors (−0.33) and medium for operated abductors (−0.59), with a relative mean decrease in strength ranging from 5% (hamstrings) to 11% (abductors) (Figure 2). The effect size (Hedges’ g) of surgery on the non-operated external rotators was small (−0.30), with a mean strength decrease of 8%.

The plasty of the acetabulum (n = 16, 55%) and of the AIIS (n = 18, 62%) did not statistically impact the muscle strength changes, probably because subgroup comparisons were statistically underpowered.

**Figure 3:**
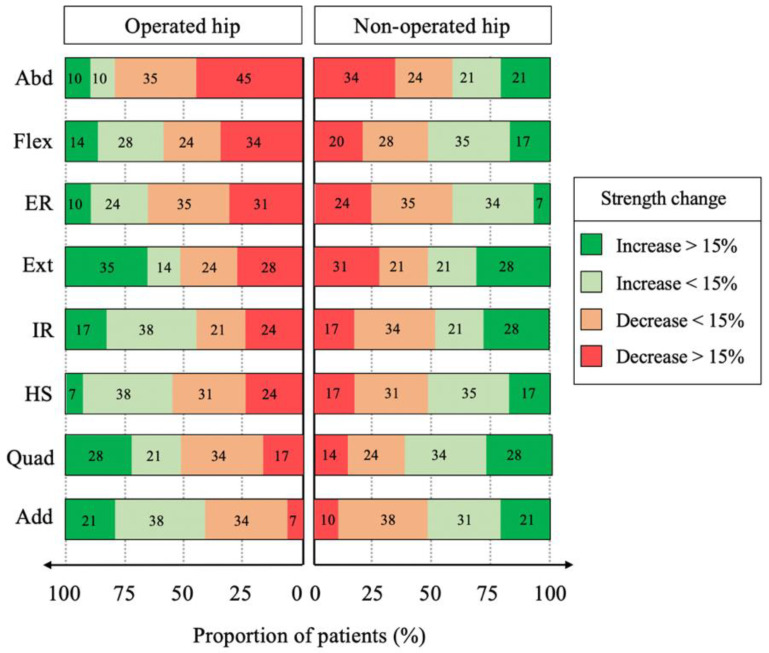
Proportion of patients with increased or decreased hip muscle strength on the operated and non-operated sides. Quadriceps (Quad), hamstrings (HS), extensor (Ext), flexors (Flex), abductors (Abd), adductors (Add), internal rotators (IR), external rotators (ER).

Among the muscles that were significantly weakened by surgery, the proportion of patients who experienced a strength reduction by 15% or more was 24% for the hamstrings, 31% for the external rotators, 34% for the flexors and 45% for the abductors on the operated side as well as 24% for the external rotators on the non-operated side (Figure 3).

**Figure 4:**
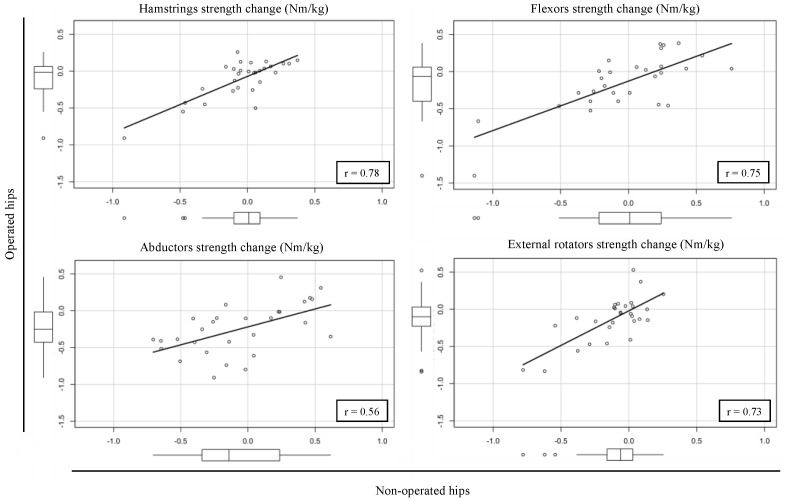
Correlation analysis of hip muscles strength change following surgery between the operated and non-operated hips.

The correlation of muscles strength change between the operated and non-operated hips was moderate for the abductors (r = 0.56; 95%CI, 0.24–0.77) and strong for the hamstrings (r = 0.78; 95%CI, 0.57–0.89), flexors (r = 0.75; 95%CI, 0.52–0.87) and external rotators (r = 0.73; 95%CI, 0.50–0.87) (Figure 4).

### 3.2. Surgical Hip Dislocation (SHD)

No intra- or postoperative complications were noted among the operated patients. Both operated and non-operated hips exhibited comparable preoperative strength on the eight tested muscles (Table 2).

While no strength changes could be noted on the non-operated side following surgery, there was a statistically significant reduction in hip muscles strength for the operated extensors (2.28 ± 0.84 vs. 2.05 ± 0.70 Nm/kg, *p* = 0.008), abductors (1.87 ± 0.49 vs. 1.65 ± 0.41 Nm/kg, *p* = 0.026), quadriceps (2.96 ± 0.92 vs. 2.44 ± 0.89 Nm/kg, *p* = 0.003), external rotators (1.16 ± 0.42 vs. 0.93 ± 0.36 Nm/kg, *p* = 0.013) and hip internal rotators (1.26 ± 0.38 vs. 0.96 ± 0.30 Nm/kg, *p* < 0.001) (Table 2).

**Figure 5:**
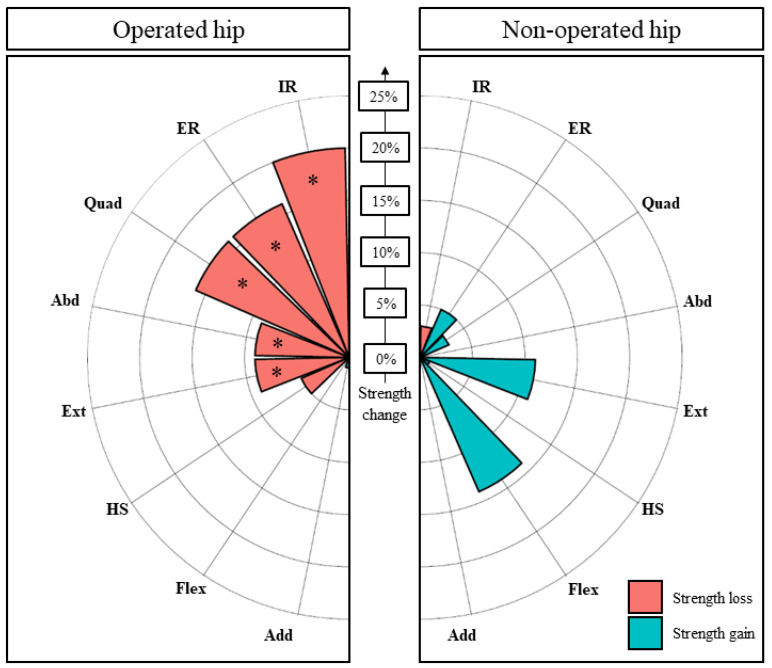
Pre- to postoperative changes (SHD) in muscle strength (%) on the operated and non-operated hips. * indicates a statistically significant variation. Internal rotators (IR), external rotators (ER), quadriceps (Quad), abductors (Abd), extensor (Ext), hamstrings (HS), flexors (Fl), adductors (Add).

The effect size (Hedges’ g) of surgery on muscle strength changes was small for extensors (−0.27), medium for abductors (−0.48), quadriceps (−0.55) and external rotators (−0.56) as well as large for internal rotators (−0.81), with a relative mean decrease in strength ranging from 9% (extensors) to 20% (internal rotators) (Figure 5). It is worth noting that the patients operated on with additional AIIS osteoplasty tended to be more affected on quadriceps strength compared to others, although this difference did not reach statistical significance (18.4% ± 22.6% vs. 5.0% ± 15.8%, *p* = 0.215).

**Figure 6:**
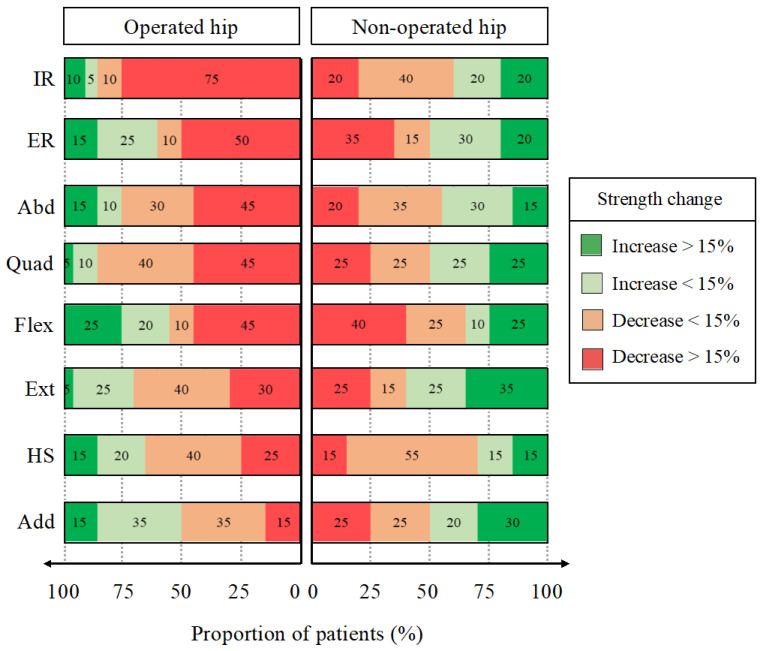
Patients distribution according to the relevance of their muscle strength change following SHD on the operated and non-operated hip. Dark green and red indicate clinically relevant variations. Internal rotators (IR), external rotators (ER), quadriceps (Quad), abductors (Abd), extensor (Ext), hamstrings (HS), flexors (Fl), adductors (Add).

Among the weakened muscles, the proportion of patients who experienced a relevant strength reduction (by 15% or more) was 30% for the extensors, 45% for the quadriceps and abductors as well as 50% and 75% for the external and internal rotators, respectively (Figure 6).

The changes in external rotator strength were strongly correlated with those exhibited by the extensors (r = 0.74, *p* < 0.001) and abductors (r = 0.71, *p* < 0.001), and the changes in extensors strength were moderately correlated with those exhibited by the abductors (r = 0.67, *p* < 0.001).

## 4. Discussion

The decision-making in the surgical treatment of FAI patients rarely relies on surgeon preferences solely. Even though arthroscopy is often considered as the gold standard treatment for FAI [7,8,9], SHD can be an adequate surgical option for patients with significant bone deformities or concomitant pathologies [10,11]. Since the aforementioned surgeries are performed on patients with different characteristics, the aim of the study was to report the strength changes in hip muscles for each procedure separately.

### 4.1. Arthroscopy

Our main results revealed a significant decrease in the strength of the abductors, external rotators, hip flexors and hamstrings on the side of the operated limb. The surgery lightly affected the hamstrings (by 5%), flexors (by 6%) and external rotators (by 7%) on the operated side. The abductors strength was the only muscle that was at least moderately affected (by 11%), with almost half of the patients having an important and relevant decrease in strength (>15%).

Muscle strength reduction on the operated side and postoperative side-to-side muscle imbalance should not be the only warning signals. Surprisingly, we observed that both operated and non-operated hips exhibited a decrease in external rotators strength following surgery. Thus, both hips are weakened without any sign of muscle strength asymmetry postoperatively. This emphasizes the fact that interpretation of strength changes following surgery in FAI patients is more complex than initially thought. Our results indicate that using the non-operated hip as a reference to better appreciate patient functional evolution over time could mislead clinicians since patients can present a contralateral hip that is also affected. Even though the underlying mechanism is not elucidated yet, this implies for clinicians specific management of the external rotators following arthroscopy with a bilateral strengthening program, as currently performed at our institution.

The mechanism behind strength reduction on the non-operated hip is currently under investigation. Several authors reported surgery to have a neurological impact involving a reorganization of the central nervous system and led to contralateral muscles strength inhibition [35,36]. Moreover, our analyses revealed, at the patient level, that the strength reductions in hamstrings, flexors and external rotators on the operated side were strongly correlated with the strength changes on the non-operated side. In other terms, it seems that patients who exhibited light or no muscle strength decrease on the operated side were those who had improved muscle strength on the non-operated side. Conversely, patients who experienced a considerable strength decrease on the operated side tended to have a strength reduction on the non-operated hip. Additional studies with greater cohort sizes should, therefore, be performed to further investigate this topic.

### 4.2. Surgical Hip Dislocation (SHD)

Our study revealed that more than half of the evaluated muscles on the operated hip exhibited a statistically significant decrease in strength at three postoperative months. The surgery lightly decreased the strength of the extensors (by 9%) and moderately reduced the strength of the abductors (by 9%), quadriceps (by 16%) and external rotators (by 16%). The most important impact was located at the internal rotators (by 20%) while affecting 75% of the patients beyond the relevant threshold of 15%. Since SHD goes along with additional soft tissue damage and trochanteric osteotomy, the authors reported different hypotheses below to explain the exhibited muscle strength changes on the operated side.

#### 4.2.1. Gluteus Medius and Minimus

The gluteus medius and minimus are the main internal rotators of the hip, with their femoral insertions located at the anterior and lateral aspects of the greater trochanter [37,38,39]. The decrease in internal rotators strength could, therefore, be explained by the osteotomy of the greater trochanter and its osteosynthesis using screws in a very intimate anatomical region. The effects of such a surgical procedure on the biomechanical properties of the gluteus medius and minimus have already been reported, thus reinforcing this hypothesis [40,41].

Likewise, the gluteus medius and minimus are important abductors of the hip, and fragilization of their femoral insertion could explain the deficit in abductor strength after surgery. The fact that the strength deficit was relatively lower in abduction (9%) than in internal rotation (20%) might be explained by the compensation of other abductor muscles that were not directly impacted by the trochanterotomy, such as the tensor fascia lata (TFL). The impact of surgery on abduction strength remains clinically relevant though as 45% of the patients had a strength decrease of more than 15% after surgery.

#### 4.2.2. Gluteus Maximus

The deficit in external rotators cannot be explained by the gluteus medius since the latter does not help in lateral hip rotation if the knee is not in extension [42]. However, a muscle that plays an important role in it is the gluteus maximus, but its insertion on the femur is not located on the greater trochanter and thus not directly impacted by the greater trochanterotomy. A hypothesis would be that screws heads on the lateral aspect of the greater trochanter irritate the gluteus maximus, thereby inducing pain and functional limitations due to repetitive movements. Given that the gluteus maximus provides most of the power used for hip external rotation, the aforementioned mechanism could explain the important decrease in external rotation strength (16%) following SHD. To put our results into perspective, this muscle strength deficit is two to three times more important than the observations made on FAI patients treated by arthroscopy [16].

Beyond its role in external rotation, the gluteus maximus also participates in hip extension and abduction (superior part of the muscle), although those movements require co-contraction of other important muscles. This can explain why those hip functions are altered but to a lesser extent owing to considerable participation of unaffected muscles located outside the surgical area (e.g., hamstrings). The involvement of the gluteus maximus in decreased hip muscles strength is also corroborated by the moderate to strong correlations observed between the strength deficit of the external rotators, extensors and abductors.

#### 4.2.3. Quadriceps

Surprisingly, quadriceps strength was considerably affected by surgery (16%), although this muscle should not be directly related to trochanteric osteotomy. A hypothesis would be that AIIS osteoplasty might have an impact on quadriceps strength but could not be verified in this study due to insufficient sample size. Further studies are, therefore, needed to investigate this hypothesis since abductors strength might have an important effect on patient progression during recovery.

### 4.3. Relevance in Clinical Practice

Postoperative strength asymmetries can generate a risk of functional imbalance and might have important consequences. They have already been reported as a risk factor for musculoskeletal injuries [43] or for intrinsic hip pathologies such as osteoarthropathy [44] while possibly affecting structures beyond the concerned joint [45]. Setting up adequate strengthening exercises in order to rectify this strength asymmetry is, therefore, essential for patients both in the short- and long-term. The exercises could initially be analytical, unilateral, combining different elaborated movements and adapted to the pain of the patient to grant specific muscles reinforcement as well as global hip function. Then, the functional imbalance could be progressively corrected by gradual loading adapted to the evolution of the symptoms.

Even though not all hip muscles were affected solely by surgery, it remains important to note that patients can present preoperative muscle weakness due to FAI syndrome, notably in abduction (11%), external rotation (18%), flexion (26%) and adduction (28%) [19,20,21]. Thus, the absence of strength reduction on the operated hip following surgery should not be necessarily considered as satisfactory. In this axis, Kierkegaard et al. [17] reported that FAI patients exhibited improved hip flexion and extension strengths one year after arthroscopy compared to pre-surgery levels; however, they remain weaker than reference persons without any hip pathology. Further studies on healthy subjects should, therefore, be performed to identify the standard strength values that clinicians could target for appropriate patient rehabilitation.

This study underlines the importance of evaluating hip muscles strength before and after surgery (SHD or arthroscopy) in order to establish the most adequate rehabilitation strategy for FAI patients. This is even more important knowing that only 2.5% of the published studies on FAI report hip muscle strengths [17,22]. Furthermore, those findings obtained halfway through the rehabilitation program clearly emphasize the importance of continuing the rehabilitation beyond three months. Finally, we now systematically propose to our SHD patients the removal of the screws once osseous union and consolidation of the greater trochanter are successful (around four months following surgery) due to their potential impact on muscle strength deterioration. Future studies evaluating the benefits of post-SHD screws removal on hip muscles strength restoration are needed.

### 4.4. Limitations

First, our sample size might not be high enough to statistically detect light muscle strength changes or the effect of surgical plasties (acetabulum, AIIS). Second, this study was only based on data collected at two different time-points and did not comprise PROMs, pain during assessments or radiological parameters. Third, our study cohort might not be comparable to FAI patients followed in other institutions. Therefore, our results might not be generalizable. Fourth, it is worth noting that small changes in muscle strength following surgery (e.g., on the non-operated external rotators after hip arthroscopy) might be related to changes in patient gait characteristics. Therefore, analyzing the gait parameters in combination with muscle strength changes would be of great interest to target the most appropriate rehabilitation program. Last, the authors did not use Bonferroni correction in their analyses since this study is exploratory and aimed at revealing potentially interesting trends [46]. Beyond the aforementioned limitations, the authors think this exploratory study adds relevant results to the existing scientific literature on the functional evolution of FAI patients following arthroscopy and SHD. An additional study analyzing the similar strength assessments following the last stage of the rehabilitation program (at seven postoperative months) will be needed to confirm whether our current practice is adequate or calls for a longer or different rehabilitation protocol for FAI patient treated by arthroscopy and SHD.

## 5. Conclusions

Our study revealed that different hip muscles on the operated FAI hip are still moderately or highly weakened 3 months after arthroscopy (abductors) or SHD (abductors, quadriceps, external and internal rotators). This emphasizes the importance of continuing rehabilitation beyond this time-point with a functional restoration program based on isolated muscle reinforcement combined with functional exercises to grant specific muscles reinforcement and global hip function. Although both treatments should not be directly compared since they concern patients with different characteristics, this study provides relevant information to clinicians on FAI patients operated on either by arthroscopy or SHD.

## Figures and Tables

**Table 1:** Pre- and postoperative hip muscles strength (Nm/kg) for arthroscopy.

	Operated Hips (n = 29)				Non-Operated Hips (n = 29)				
	Mean	±SD	Median	IQR Range	Mean	±SD	Median	IQR Range	*p*
**Quadriceps**									
Preoperative	2.72	±0.69	2.65	(2.45–3.11)	2.82	±0.77	2.77	(2.50–3.26)	0.177
Postoperative	2.69	±0.52	2.62	(2.43–3.06)	2.86	±0.69	2.90	(2.34–3.26)	0.095
Change (%)	2%	±19%	−1%	(−9–15%)	4%	±19%	5%	(−5–15%)	
*p*-value	0.781				0.581				
**Hamstrings**									
Preoperative	1.49	±0.43	1.48	(1.18–1.79)	1.49	±0.46	1.49	(1.15–1.83)	0.932
Postoperative	1.39	±0.38	1.38	(1.15–1.64)	1.45	±0.36	1.44	(1.16–1.63)	0.064
Change (%)	−5%	±16%	−1%	(−14–6%)	1%	±19%	1%	(−6–7%)	
*p*-value	0.039				0.380				
**Extensors**									
Preoperative	2.11	±0.60	2.16	(1.74–2.45)	2.10	±0.53	2.06	(1.84–2.39)	0.946
Postoperative	2.14	±0.72	2.19	(1.66–2.59)	2.10	±0.59	2.05	(1.88–2.48)	0.661
Change (%)	4%	±30%	0%	(−19–19%)	2%	±26%	2%	(−17–19%)	
*p*-value	0.752				0.997				
**Flexors**									
Preoperative	1.88	±0.46	1.84	(1.67–2.02)	1.95	±0.50	1.86	(1.68–2.08)	0.132
Postoperative	1.73	±0.41	1.67	(1.48–1.90)	1.93	±0.42	1.90	(1.57–2.22)	0.012
Change (%)	−6%	±18%	−5%	(−20–3%)	1%	±20%	0%	(−12–13%)	
*p*-value	0.046				0.865				
**Abductors**									
Preoperative	1.97	±0.42	2.06	(1.78–2.23)	1.98	±0.45	1.96	(1.65–2.37)	0.804
Postoperative	1.72	±0.40	1.69	(1.49–2.05)	1.92	±0.34	1.95	(1.76–2.12)	0.001
Change (%)	−11%	±18%	−13%	(−22–1%)	0%	±23%	−6%	(−18–13%)	
*p*-value	<0.001				0.394				
**Adductors**									
Preoperative	1.81	±0.54	1.73	(1.58–2.07)	1.90	±0.49	1.88	(1.65–2.14)	0.393
Postoperative	1.86	±0.45	1.85	(1.57–2.17)	1.88	±0.44	1.86	(1.59–2.26)	0.680
Change (%)	6%	±21%	4%	(−4–9%)	2%	±19%	4%	(−7–13%)	
*p*-value	0.340				0.417				
**Internal rotators**									
Preoperative	1.13	±0.37	1.05	(0.86–1.28)	1.14	±0.43	1.04	(0.84–1.25)	0.776
Postoperative	1.11	±0.33	1.10	(0.91–1.30)	1.15	±0.38	1.10	(0.94–1.47)	0.249
Change (%)	1%	±23%	5%	(−11–13%)	4%	±21%	0%	(−7–15%)	
*p*-value	0.709				0.811				
**External rotators**									
Preoperative	1.17	±0.40	1.09	(0.90–1.53)	1.21	±0.38	1.15	(0.94–1.48)	0.177
Postoperative	1.04	±0.37	0.92	(0.82–1.14)	1.10	±0.36	1.04	(0.88–1.33)	0.190
Change (%)	−7%	±25%	−7%	(−22–4%)	−8%	±17%	−6%	(−13–3%)	
*p*-value	0.022				0.014				

SD, standard deviation; IQR, interquartile range; *p*, *p*-value.

**Table 2:** Pre- and postoperative hip muscles strength (Nm/kg) for surgical hip dislocation.

	Operated Hips (n = 20)				Non-Operated Hips (n = 20)				
	Mean	±SD	Median	IQR Range	Mean	±SD	Median	IQR Range	*p*
**Quadriceps**									
Preoperative	2.96	±0.92	2.88	(2.48–3.46)	3.00	±0.81	2.98	(2.55–3.63)	0.744
Postoperative	2.44	±0.89	2.56	(1.72–2.94)	3.08	±1.05	2.99	(2.36–3.81)	<0.001
Change (%)	−16%	±22%	−11%	(−25–1%)	3%	±26%	2%	(−15–11%)	
*p*-value	0.003				0.635				
**Hamstrings**									
Preoperative	1.52	±0.38	1.45	(1.23–1.74)	1.45	±0.38	1.38	(1.24–1.72)	0.139
Postoperative	1.44	±0.41	1.50	(1.13–1.72)	1.53	±0.43	1.56	(1.24–1.85)	0.045
Change (%)	−5%	±20%	−7%	(−15–6%)	11%	±44%	8%	(−4–11%)	
*p*-value	0.182				0.240				
**Extensors**									
Preoperative	2.28	±0.84	2.41	(1.80–2.71)	2.31	±0.88	2.24	(1.65–2.94)	0.775
Postoperative	2.05	±0.70	2.27	(1.47–2.47)	2.26	±0.77	2.34	(1.98–2.62)	0.016
Change (%)	−9%	±14%	−10%	(−16–1%)	−0%	±27%	−4%	(−19–9%)	
*p*-value	0.008				0.662				
**Flexors**									
Preoperative	2.08	±0.75	1.89	(1.47–2.78)	2.14	±0.70	2.14	(1.55–2.54)	0.415
Postoperative	2.01	±1.02	1.86	(1.53–2.29)	2.36	±0.88	2.28	(1.72–2.79)	0.015
Change (%)	0%	±39%	−3%	(−21–14%)	14%	±34%	12%	(−14–32%)	
*p*-value	0.571				0.198				
**Abductors**									
Preoperative	1.87	±0.49	1.73	(1.60–2.09)	1.98	±0.51	1.80	(1.62–2.15)	0.097
Postoperative	1.65	±0.41	1.61	(1.36–1.93)	1.95	±0.59	2.02	(1.64–2.26)	0.015
Change (%)	−9%	±22%	−13%	(−22–1%)	−0%	±25%	2%	(−5–14%)	
*p*-value	0.026				0.498				
**Adductors**									
Preoperative	2.05	±0.68	1.87	(1.56–2.78)	2.09	±0.71	1.75	(1.57–2.84)	1.000
Postoperative	2.02	±0.66	2.12	(1.58–2.46)	2.02	±0.45	2.07	(1.64–2.36)	0.989
Change (%)	1%	±23%	1%	(−11–8%)	1%	±18%	0%	(−16–13%)	
*p*-value	0.730				0.522				
**Internal rotators**									
Preoperative	1.26	±0.38	1.29	(1.06–1.50)	1.26	±0.43	1.22	(1.01–1.47)	0.900
Postoperative	0.96	±0.30	0.87	(0.74–1.15)	1.29	±0.47	1.26	(1.03–1.41)	<0.001
Change (%)	−20%	±22%	−25%	(−32–13%)	−3%	±17%	4%	(−9–12%)	
*p*-value	<0.001				0.546				
**External rotators**									
Preoperative	1.16	±0.42	1.06	(0.88–1.49)	1.10	±0.36	1.02	(0.84–1.38)	0.171
Postoperative	0.93	±0.36	0.87	(0.70–1.13)	1.15	±0.43	1.14	(0.80–1.33)	0.003
Change (%)	−16%	±25%	−14%	(−31–4%)	5%	±24%	0%	(−11–24%)	
*p*-value	0.013				0.496				

SD, standard deviation; IQR, interquartile range.

## Data Availability

Data supporting the reported results can be requested from the corresponding author (hugo.bothorel@latour.ch).

## Supplementary Materials

The following supporting information can be downloaded at: https://www.mdpi.com/article/10.3390/biology11121765/s1, Text S1: Strength testing positions.
