# Supplementary material for: Evolution of Hip Muscles Strength in Femoroacetabular Impingement Patients Treated by Arthroscopy or Surgical Hip Dislocation: A Retrospective Exploratory Study
Source: Biology (Basel). 2022 Dec 6;11(12):1765. doi: 10.3390/biology11121765 (PMC9775450; doi:10.3390/biology11121765)
Supplement: Supplementary file 1 [file biology-11-01765-s001.zip › biology-2071811-supplementary.pdf]

Supplementary Material (Text S1): Strength testing positions.

*Note: a fixation-belt was systematically used. (1) Hip abductors: leg tested: in extension, placed on the table, respect the alignment of the segment. Contralateral leg: Knee bent, foot resting on the table. The belt placed at ankle level, perpendicular to the anatomical segment, the strap is placed around the table: (2) Hip adductors: same position as for the hip abductors by reversing the direction of the resistance. (3) Hip external rotators: Patient seated at the edge of the table with his feet in the air, vertical legs, popliteal fossa a finger's breadth from the edge of the table. The belt placed above the malleolus, perpendicular to the anatomical segment with fixed point on physiotherapist leg. (4) Hip internal rotators: same position than the external rotators except the resistance leg side. (5) Hip flexors: Leg tested: Patient seated at the edge of the table with the feet in the air, knees at 90°, popliteal fossa a fingers' breadth from the edge of the table. The belt placed two fingers' breadths above the patella, the strap is fixed under the foot of the physiotherapist. Placement of the dynamometer: two fingers' breadths above the patella. (6) Hip extensors: The belt goes around the table and passes through fingers above the popliteal fossa of the tested leg and under the untested leg. Dynamometer placement: three fingers' breadths above the popliteal fossa. (7) Quadriceps: Leg tested: Patient seated at the edge of the table with the feet in the air, knees at 90°, popliteal fossa at a finger breadth from the edge of the table. Above the malleolus, perpendicular to the anatomical segment to be measured with a fixed point on the foot of the table. Placement of the dynamometer: On the front side of the leg, two fingers' breadths above the malleolus. (8) Hamstrings: same position than the external rotators except the resistance leg side.
